# Retraction: Spontaneous pre-stimulus fluctuations in the activity of right fronto-parietal areas influence inhibitory control performance

**DOI:** 10.3389/fnhum.2013.00923

**Published:** 2013-12-27

**Authors:** Frontiers in Neuroscience Editorial

The authors and the journal wish to retract the 12 March 2013 article cited above.

While applying the same analyses to another dataset, the authors discovered that a systematic human error in coding the name of the files had been made during the extraction of the EEG template topographic maps best differentiating the two experimental conditions at the single subject level. Because the subsequent processing steps were based on these EEG maps, this error has ultimately modified the final result of the paper, which is therefore not correct. For this reason, the authors request to withdraw the article.

All the authors concur with this retraction and sincerely regret any inconvenience this may have caused to the reviewers, editors, and readers of Frontiers in Human Neuroscience.

